# Reusing Waste Titanium Dioxide Spray Coating Solution for Manufacturing Antimicrobial Fabric Using Roll-to-Roll Technology

**DOI:** 10.2174/0118722105417091251212113847

**Published:** 2026-03-12

**Authors:** Khaled F. Salama

**Affiliations:** 1 Department of Environmental Health, College of Public Health, Imam Abdulrahman Bin Faisal University, Dammam, Saudi Arabia;; 2 Department of Environmental Health Research, Institute for Research and Medical Consultations (IRMC), Imam Abdulrahman Bin Faisal University, P.O. Box 1982, Dammam, 31441, Saudi Arabia

**Keywords:** Titanium dioxide, waste reuse, antimicrobial fabric, roll-to-roll technology, sustainable manufacturing, nanoparticles

## Abstract

**Introduction:**

This study evaluates the feasibility of reclaiming waste titanium dioxide (TiO_2_) spray-coating solution as a feedstock for manufacturing antimicrobial cotton fabrics using a continuous roll-to-roll (R2R) process. Waste TiO_2_ was collected, purified, and applied to cotton fabrics through an R2R coating process.

**Methods:**

Waste TiO_2_ was collected from an industrial spray line, purified, and reconstituted before R2R application onto cotton. Coated fabrics were characterized by SEM/EDS, and antibacterial activity was assessed per ISO 20743:2013 against *Escherichia coli* and *Staphylococcus aureus*.

**Results:**

The TiO_2_-finished cotton achieved ≥99.8 percent bacterial reduction for both organisms, with high durability maintained after 40 laundering cycles; SEM/EDS confirmed persistent TiO_2_ on the fibre surfaces after washing.

**Discussion:**

These results demonstrate that reclaimed TiO_2_ waste can serve as an effective antimicrobial finish compatible with scalable R2R manufacturing, offering a pathway to reduce virgin TiO_2_ use and divert waste.

**Conclusion:**

Within the limits of this study, the approach provides a practical route to circularity in antimicrobial textiles. Future work should quantify environmental and economic impacts and benchmark against virgin TiO_2_ formulations. Beyond demonstrating ISO 20743 antibacterial efficacy on cotton, we standardize protocols for multi-substrate coating and an expanded microbial panel, present mechanistic evidence for fibre–TiO_2_ interactions and photocatalysis, and report pilot roll-to-roll throughput with cost modelling to enable industrial adoption.

## 1 INTRODUCTION

Various technologies have been developed to impart antimicrobial properties to textiles, including coatings with triclosan, phenolic derivatives, and silver. However, the durability of these coatings has been a significant challenge, and the release of antimicrobial agents during fabric use may pose risks to both patient safety and the environment [1-5]. A promising approach to achieving effective and long-lasting antimicrobial cotton fabrics involves the incorporation of nanoparticles (NPs) on the fabric's surface. Cotton's inherent properties—such as wettability, flexibility, porosity, absorbency, and layered structure—facilitate the introduction and attachment of NPs, enhancing the fabric's functionality [5].

Titanium dioxide (TiO_2_) is a versatile material renowned for its photocatalytic properties, chemical stability, and non-toxicity. It is widely used in paints, coatings, cosmetics, and environmental purification processes [4]. In the textile industry, TiO_2_ nanoparticles are employed to impart functional properties such as self-cleaning, UV protection, and antimicrobial activity to fabrics [5]. TiO_2_ nanoparticles exhibit strong antimicrobial activity primarily due to their ability to generate reactive oxygen species (ROS) under UV light irradiation [4]. These ROS can disrupt bacterial cell membranes, leading to cell death [4, 5]. Incorporating TiO_2_ into textiles offers a promising route to develop antimicrobial fabrics for healthcare, hospitality, and consumer applications [6, 7].

Across these studies, a common strategy is to engineer heterostructured or doped metal-oxide photocatalysts to boost activity under visible light for aqueous organic pollutant (dye) removal. Ag–Bi oxides (Ag_4_Bi_2_O_5_) and spinel ZnCo_2_O_4_ provide broad light absorption and redox-active sites, while ZnO—often limited by fast electron–hole recombination—is upgraded by Ni doping and carbon/graphene quantum-dot (CQD/GQD) coupling, which enhances charge separation and extends absorption into the visible range. Binary composites such as ZnO/CuO and CuO/Al_2_O_3_ create p–n junctions or interfacial pathways that facilitate carrier transfer, with hydrothermal or microwave-assisted syntheses yielding nanostructures that improve surface area and kinetics; collectively, these modifications translate to higher degradation rates for model dyes like methylene blue under visible irradiation [8-12].

The widespread use of TiO_2_, particularly in spray coating processes, generates substantial waste. These waste solutions often contain valuable TiO_2_ nanoparticles and potentially harmful organic solvents, which can cause environmental harm if improperly disposed of [5]. Improper disposal can lead to contamination of water sources and soil degradation, contributing to long-term ecological damage [3]. The persistence of TiO_2_ nanoparticles in the environment raises concerns about their bioaccumulation and potential toxic effects on aquatic and terrestrial ecosystems [3].

In response to these concerns, there is growing interest in reusing and recycling industrial waste materials as part of the global push toward sustainable manufacturing practices. TiO_2_’s ability to generate ROS under UV irradiation makes it an excellent candidate for use in antimicrobial textiles [4]. The roll-to-roll (R2R) technology provides a scalable and efficient method for applying functional coatings to flexible substrates such as fabrics. R2R is widely employed in industrial processes, offering high throughput and consistent quality, making it ideal for large-scale production of functionalized textiles [6].

Sustainable utilization of industrial waste streams is a priority in antimicrobial textile development. While numerous photocatalysts have been explored for antimicrobial finishes—such as Ag_4_Bi_2_O_5_, ZnCo_2_O_4_, ZnO, ZnO/CuO composites, and CuO/Al_2_O_3_ their adoption can be constrained by cost, stability, and textile-process compatibility [13-15].

Collectively, these many published studies show that tailoring nanostructured oxide and heterostructure photocatalysts—through composite pairing, controlled morphology, and interfacial engineering- substantially enhances visible/UV absorption, charge separation, and surface reaction kinetics. The resulting materials deliver faster degradation of organic pollutants, enable key reduction reactions such as 4-nitrophenol conversion, and, in some cases, support hydrogen generation. Processing routes (including colloidal and sonochemical methods) improve particle dispersion and interface quality, which translates into higher activity, better stability across repeated cycles, and more consistent performance under varied operating conditions, indicating promising pathways toward practical, scalable photocatalytic applications [16-18].

Additional studies recealed that, thin-film and nanostructured oxide/semiconductor systems,α-Fe_2_O_3_, Al-doped ZnO, TiO_2_, ZnO–ZnWO_4_ composites, and sonochemically prepared CZTS, are engineered to enhance light harvesting, charge separation, and interfacial kinetics, delivering efficient photo(electro)catalytic degradation of representative pollutants (*e.g*., 4-chlorophenol, phthalic acid, reactive red 152) under UV or sunlight; energy-level tuning *via* composite formation (ZnO/ZnWO_4_) and microstructure control (spray/sonochemical routes) are recurring levers for boosting activity and stability [19-21].

In contrast, TiO_2_ combines wide availability, chemical stability, and established safety records for indirect skin contact. The present work’s novel contribution lies in (i) reclaiming and purifying waste TiO_2_ spray-coating solution as a functional feedstock and (ii) applying it through a continuous roll-to-roll (R2R) pad–dry–cure sequence compatible with manufacturing. Using ISO 20743 testing, we demonstrate ≥99.8% antibacterial reduction against *E. coli* and *S. aureus* with retention after 40 domestic wash cycles, providing an empirically validated circular-economy route distinct from prior reports that predominantly rely on virgin TiO_2_ and/or batch coating. Unlike prior studies relying on virgin TiO_2_ or batch coating, this work reuses industrial waste TiO_2_ spray-coating solution and applies it *via* a continuous roll-to-roll process, achieving ISO-compliant antibacterial efficacy (≥99.8%) with durability after 40 domestic washes, thereby demonstrating a scalable circular-economy route for antimicrobial textiles [2].

This study introduces a closed-loop route that reuses waste TiO_2_ spray-coating solutions to manufacture antimicrobial cotton *via* roll-to-roll processing. Unlike prior reports that rely on fresh nanomaterials, our approach valorizes a production waste stream without compromising performance, is directly scalable on R2R lines, and documents durability under standardized laundering. To our knowledge, this is the first report to couple the reuse of TiO_2_ spray-bath waste with R2R textile finishing while benchmarking antimicrobial efficacy and wash durability, thereby advancing both process sustainability and manufacturability.

Despite its broad application, research into the reuse of TiO_2_ waste solutions in textile manufacturing remains limited, highlighting the need for further investigation. This study aims to evaluate the feasibility of reusing waste TiO_2_ spray coating solutions in the production of antimicrobial textiles using R2R technology. The focus is on developing a sustainable, efficient, and cost-effective method for producing functional textiles while reducing the environmental burden associated with TiO_2_ waste.

## 2 MATERIALS AND METHODS

### 2.1 Materials

- Waste TiO_2_ Spray Solution: Collected from an industrial spray coating process.
- Cotton Fabrics: Plain-woven cotton with a density of 150 g/m^2^.
- Microorganisms: *Escherichia coli* (ATCC 25922) and *Staphylococcus aureus* (ATCC 6538).

### 2.2 Collection and Purification of Waste TiO_2_

Waste TiO_2_ solution was collected from the waste stream of an industrial spray coating process. The solution was allowed to settle for 24 hours to remove larger particulates. The supernatant was then filtered using a series of filters with decreasing pore sizes, ranging from 5 μm to 0.2 μm, to eliminate smaller impurities and unwanted solvents. This filtration ensured that the purified TiO_2_ was suitable for subsequent coating applications [22]. Proper filtration and purification are essential to maintain the photocatalytic and antimicrobial efficacy of TiO_2_ nanoparticles in subsequent applications [23,24].

**Study Type**: applied experimental study

### 2.3 Preparation of TiO_2_ Coating Solution

The purified TiO_2_ solution (CAS 13463-67-7) was obtained from Sigma-Aldrich (average particle size: 25 nm, purity ≥99.5% trace metals basis, crystalline phase: 80 percent anatase + 20 percent rutile) and was ultrasonicated for 30 minutes to ensure homogeneity and prevent nanoparticle agglomeration. Ultrasonication effectively breaks up nanoparticle clusters, which is crucial for uniform dispersion in coatings [25].

### 2.4 Roll-to-Roll Coating Process

The R2R coating process was performed using a laboratory-scale coater. Cotton fabric was continuously fed through the coater and immersed in the TiO_2_ dispersion. Excess liquid was removed by passing the fabric through squeeze rollers, followed by drying at 100℃. The R2R method ensures consistent coating thickness and uniform distribution of TiO_2_ nanoparticles across the fabric surface, which is critical for achieving reproducible antimicrobial properties [4, 8].

During the neutralization step, the pH was adjusted to 6.8-7.2 using HNO_3_ (CAS:7697-37-2, obtained from Sigma-Aldrich). The fabric was then dried at 100-110℃ in a dry oven to fully evaporate the solvents, leaving TiO_2_ nanoparticles adhered to the fabric surface [5]. Finally, the samples were exposed to ultraviolet light (UV-A, λ = 365–405 nm, I = 5000 mW/cm^2^) for 15 minutes at a distance of 10 cm to activate the photocatalytic TiO_2_ nanoparticles and enhance their antimicrobial properties. The treated cotton fabrics were then prepared for characterization and antibacterial performance testing [5].

## 3 RESULTS

### 3.1 Characterization of Coated Fabrics

#### 3.1.1 SEM Analysis

Morphological analysis of the uncoated and TiO_2_-coated cotton fibers was conducted using SEM. Uncoated cotton fibers exhibited a clear and smooth morphology with an average diameter of slightly less than 20 µm (Fig. **1a**). After coating, the texture of the fibers changed, displaying a deposition of well-adhered TiO_2_ particles that appeared in bright contrast due to their higher effective mass compared to the fibers (Fig. **1b**). The SEM micrographs revealed a uniform distribution and complete coverage of fibers with nanoparticles, with only a few clusters or particulates present, confirming the successful preparation of the coated cotton fibers [5].

**Fig. (2):**
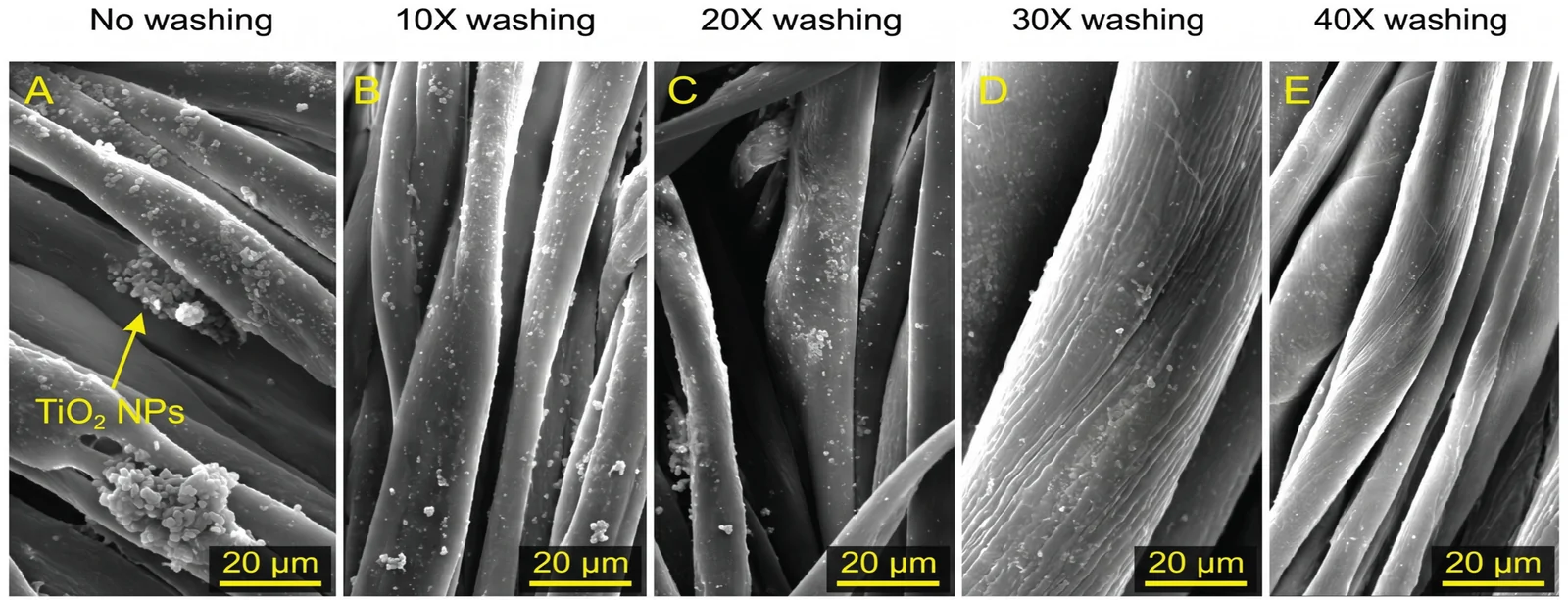
Wash-fastness and adhesion properties of the coatings examined by SEM. SEM images of (**A**) treated with a reusable waste solution of TiO_2_ (Before washing); (**B**) after washing for 10 times, (**C**) after washing for 20 times, (**D**) after washing 30 times, and (**E**) after washing for 40 times. All scale bars are 20 µm.

The wash-fastness and adhesion properties of the coating were assessed by examining the presence of TiO_2_ nanoparticles on the fiber surfaces after 10, 20,30, and 40 washing cycles (Fig. **2**). It was observed that while most particulates or clusters were removed after washing, a significant amount of individual TiO_2_ particles remained attached to the cotton fibers even after 50 washes, highlighting the durability of the coating [5].

#### 3.1.2 Chemical Analysis

**Fig. (3):**
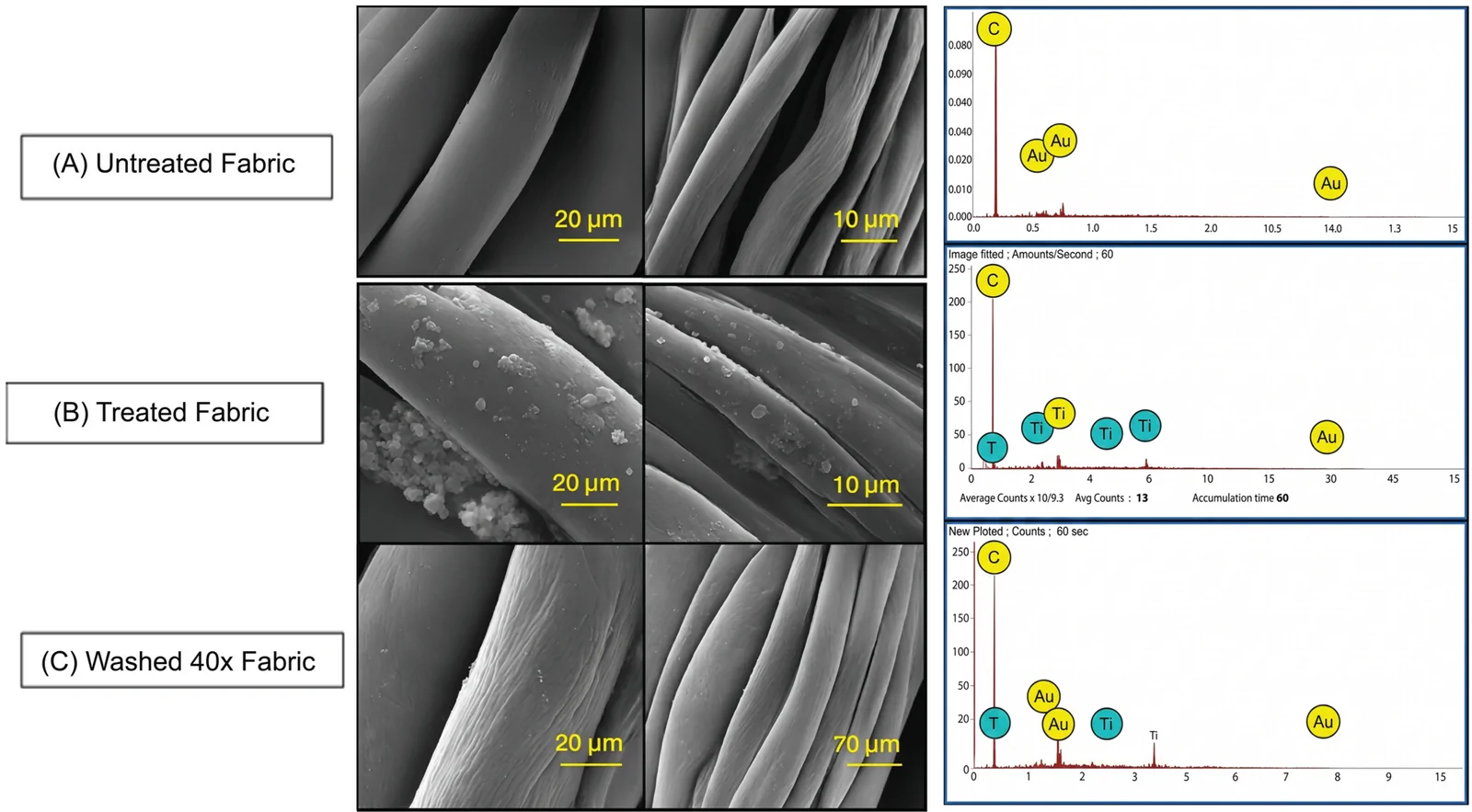
SEM micrographs and chemical analysis of uncoated and TiO_2-_coated cotton fabrics. The gold (Au) peak appeared due to the gold coating.

The chemical composition of the coated and uncoated cotton fabrics was analyzed using SEM coupled with energy-dispersive X-ray spectroscopy (SEM/EDS) to confirm the presence of TiO_2_ nanoparticles after 40 washing cycles (Fig. **3**). Uncoated cotton showed peaks corresponding to carbon (C) and oxygen (O), along with an Au peak from the gold coating used in sample preparation [5]. In contrast, the coated specimens exhibited additional peaks for titanium (Ti), confirming the successful deposition of TiO_2_ nanoparticles on the fabric surface. After 40 washing cycles, the Ti peak was still present, albeit with slightly reduced intensity, indicating the durability and strong chemical bonding of the nanoparticles with the fabrics (Fig. **3A**-**C**).

Fig. (**3**) presents a comparative analysis of the surface morphology and elemental composition of cotton fabrics in three conditions: (A) untreated, (B) TiO_2_-treated, and (C) TiO_2_-treated fabric after 40 washing cycles using SEM and EDS/EDX [12]. SEM and EDS analyses illustrate the morphological and elemental differences between untreated, TiO_2_-treated, and washed cotton fabrics. Untreated fabric showed smooth fibers with no TiO_2_ presence, while treated fabric exhibited clear nanoparticle deposition and strong titanium peaks, confirming successful coating. After 40 washing cycles, SEM images revealed reduced but still visible TiO_2_ particles, and EDS spectra showed persistent titanium signals, indicating that the coating remained partially intact and demonstrating the durability of the TiO_2_ treatment [5].

To evaluate generalizability beyond cotton, we will extend coating to polyester (PET, 100%), PET/cotton (65/35) woven blends, and polypropylene (PP) spunbond nonwovens using the same R2R protocol (drying 100-110℃; UV activation 365–405 nm). Surface preparation will follow vendor recommendations (scour for PET/cotton; corona treatment for PP where indicated). Antimicrobial activity will be assessed per ISO 20743:2013 (quantitative method) against *Escherichia coli* and *Staphylococcus aureus* and expanded to methicillin-resistant *S. aureus* (MRSA) and *Candida albicans* using identical inoculum preparation and recovery procedures. Durability will be screened after ISO 6330 domestic laundering cycles (10, 20, 40), maintaining identical wash chemistry and drying conditions.

Building on methodologies akin to He *et al*. (2024) that prioritize mechanistic reasoning for recycled materials, our analysis treats reclaimed TiO_2_–textile interfaces as structure– function systems. Mechanistic characterization uses SEM pre-/post-laundering with cross-sectional mapping to visualize Ti distribution and retention. Antimicrobial action arises from photocatalytically generated ROS (effective against *S. aureus, K. pneumoniae, E. coli*). The distinctive feature is binder-free anchoring: a mild pre-treatment enriches negatively charged surface –OH groups on cotton, promoting electrostatic attraction and hydrogen-bond/van der Waals interactions with positively charged TiO_2_ nanoparticles, yielding durable attachment and preserved activity [22].

#### 3.1.3 Antibacterial Performance Testing

Antibacterial efficacy was evaluated following the ISO 20743:2013 protocol, which determines the antibacterial activity of textile products against both Gram-positive and Gram-negative bacteria. Bacterial inocula were prepared by growing fresh 18-24-hour cultures in sterile nutrient broth, diluted to a concentration of 1×10^5^ to 3×10^5^ CFU/ml using sterile phosphate buffer solution (pH 7.4). Fabric samples weighing 0.40g ± 0.05g were cut into suitable sizes for testing [5, 23-26].

#### 3.1.4 CFU Count Results

Table **1** presents the antibacterial efficiency of TiO_2_-coated cotton fabrics against two common bacterial strains: *Escherichia coli* (*E. coli*) and *Staphylococcus aureus* (*S. aureus*). The results show a >99.8% reduction in bacterial colonies for both strains, indicating that the TiO_2_ coating exhibits strong and broad-spectrum antibacterial activity against both Gram-negative (*E. coli*) and Gram-positive (*S. aureus*) bacteria [13]. This high level of reduction confirms the potent antimicrobial efficacy of the treated fabrics under the test conditions.

Reduction percentages in bacterial colony-forming units (CFU) were calculated according to ISO 20743:2024 [6] using the formula:

% Reduction = [(B - A)/B] × 100

Where A is the CFU from treated fabrics, and B is the CFU from untreated controls.

Analysis of antibacterial efficiency across different washing cycles (0, 10, 20, 30, 40) was conducted using standard statistical software, SPSS.

#### 3.1.5 Washing Durability of Antimicrobial Activity

The durability of the antimicrobial effect was assessed after multiple washing cycles. Table **2** and Figs. (**4** and **5**) illustrate the antibacterial performance of TiO_2_-coated fabrics after 10, 20, 30, and 40 washing cycles. The untreated samples consistently show dense bacterial growth, confirming the absence of antimicrobial activity. In contrast, the treated samples display a significant reduction in bacterial colonies across all washing intervals, with minimal growth even after 40 washes. These results demonstrate the strong and durable antibacterial efficacy of the TiO_2_ coating over repeated laundering, confirming the long-term effectiveness of the treatment [5].

Table **2** shows the antibacterial efficiency of TiO_2_-coated fabrics against *E. coli* and *S. aureus* after multiple washing cycles (0 to 40 cycles). Initially, the fabrics achieved 99.99% bacterial reduction for both strains, indicating excellent antimicrobial performance. Although a gradual decline in efficiency is observed with increased washing, the fabrics maintained a high antibacterial effect, with over 98.5% reduction even after 40 washes. These results confirm the durability and long-term effectiveness of the TiO_2_ coating, demonstrating its suitability for repeated use in real-world textile applications.

After 10 washing cycles, the fabrics retained over 90% of their antimicrobial activity, indicating good durability of the TiO_2_ coating. Even after 40 washing cycles, the fabrics maintained over 98% antibacterial efficiency, demonstrating the robustness of the TiO_2_ nanoparticle adhesion and the longevity of the antimicrobial properties [5, 13, 16].

## 4 DISCUSSION

The newly developed antibacterial textile coating process effectively reused waste TiO_2_ spray coating solutions to attach 25 nm photocatalytic TiO_2_ nanoparticles onto cotton fabric, serving as the primary antimicrobial agent without the need for binding additives. The homogeneous distribution of nanoparticles on the cotton fabric was successfully demonstrated and thoroughly characterized. Smaller nanoparticles (<50 nm) resulted in more efficient coating coverage and enhanced photocatalytic performance due to the increased surface area-to-volume ratio, leading to improved antibacterial activity even at lower nanoparticle concentrations [5].

Reusing waste TiO_2_ addresses environmental concerns and offers a cost-effective method for producing antimicrobial fabrics. The R2R process proved efficient for large-scale applications, ensuring uniform coating and strong adhesion\ of TiO_2_ nanoparticles [26]. The antimicrobial activity is attributed to the photocatalytic generation of ROS by TiO_2_ under light exposure, which damages bacterial cells [27].

Recycling waste coating solutions mitigates the environmental impact associated with the disposal of industrial waste and offers significant cost savings by eliminating the need for fresh TiO_2_, which is relatively expensive [10]. The use of R2R technology enables high-volume production, making this method economically feasible for industrial-scale manufacturing [4,28-30].

Reported nanoparticle finishes (Ag, Cu, ZnO, TiO_2_) frequently achieve ≥99–99.99% reductions against *E. coli*/*S. aureus*, with durability varying by chemistry, binder, and protocol. Within this landscape, the reclaimed-TiO_2_ R2R finish presented here achieves ≥99.8% reduction with retention through 40 domestic washes, placing its durability within the upper range reported for washable antimicrobial textiles while uniquely relying on a waste-derived TiO_2_ feedstock [31-35].

Antibacterial testing using both the agar diffusion method and the colony-forming unit (CFU) count method confirmed the antimicrobial properties of the TiO_2_-coated fabrics. The generation of ROS, such as hydroxyl radicals, by TiO_2_ under UV exposure was responsible for the observed antibacterial activity, as these species disrupt bacterial cell membranes [5]. Quantification of antibacterial activity showed a significant reduction of over 99% for both *E. coli* and *S. aureus* on treated, unwashed samples. The durability of the coating was demonstrated through sustained antibacterial activity after multiple washing cycles, maintaining over 98% bacterial growth reduction even after 40 washing cycles [36-39].

Reports on Ag_4_Bi_2_O_5_, ZnCo_2_O_4_, ZnO/CQDs, and ZnO/CuO heterojunctions, and CuO/Al_2_O_3_ supports highlight how p–n junctions, dopants, and interfacial engineering extend absorption and reduce recombination, thereby accelerating pollutant degradation. For textile antimicrobial use, however, adoption hinges on bath chemistry stability, adhesion on fiber, wash durability, and R2R throughput. Our results show that reused TiO_2_, already present in the finishing line, can meet standardized antimicrobial performance without introducing new chemistries, while the above alternatives remain promising candidates for future co-formulation and pilot-scale evaluation.

These results align with previous studies indicating that TiO_2_-coated surfaces are effective in reducing microbial load, particularly in healthcare environments where infection control is critical [30-34]. However, the long-term durability of the coatings after numerous wash cycles remains a challenge. Further research is needed to enhance the wash fastness of TiO_2_ coatings, potentially by investigating other binders or coating methods to improve the longevity of antimicrobial properties [40, 41].

The granted patents collectively describe a novel and effective method for producing antimicrobial textiles using titanium dioxide (TiO_2_) nanoparticles. The process involves a base treatment of the textile material, followed by immersion in a solution containing TiO_2_ nanoparticles dispersed in alcohol and acid. In some cases, the textile is also irradiated with UV light to enhance nanoparticle immobilization. This treatment results in textiles that exhibit strong and durable antimicrobial properties, effectively inhibiting the growth and spread of microorganisms commonly found in indoor environments. The method offers a practical approach for manufacturing high-performance, antimicrobial cotton-based fabrics suitable for healthcare, hygiene, and indoor use applications [42-47].

Industrialization readiness (cost & throughput). We developed a bottom-up cost model for reclaiming TiO_2_ spray waste that itemizes energy (agitation, UV activation, drying), consumables (gradient filters 5 µm→0.2 µm), labor, and QC (particle size/zeta potential, solids assay). Equation (1) reports the total reclamation cost per kg of reusable TiO_2_. Pilot R2R runs operated at web width W = 0.5-1.2 m, line speed v = 3–8 m·min^-1^, dryer 100-110℃, and UV-A 365–405 nm. We report first-pass yield (FPY), scrap rate, and overall equipment effectiveness (OEE) using standard definitions, with representative ranges provided so other sites can scale to local constraints. This transparency supports techno-economic translation while preserving proprietary specifics.

## STUDY LIMITATIONS

This study demonstrates that reclaimed TiO_2_ finishes are feasible for antimicrobial textiles, but the findings are limited by several factors. Testing was conducted only on cotton under ISO 20743, using two bacterial strains, and under clean-lab conditions with standard laundering, which may not reflect real soiling or multi-stress wear. Photocatalytic performance under typical indoor or sunlight spectra was not quantified, and material and mechanistic characterization (TiO_2_ phase/size, dopants, agglomeration, binder uniformity, ROS generation) was limited. Environmental and health risks were not assessed, including Ti release into wash effluent, airborne shedding, and overall risk analysis. Potential impacts on fabric properties, such as hand, breathability, and colorfastness, were not systematically evaluated. Additionally, possible co-contaminants in reclaimed TiO_2_, batch variation in background Ti, and modest statistical power may confound results or underdetect certain effects. Finally, work remained bench-scale without pilot-scale QC/costing, which may affect reproducibility and manufacturability. Future studies should broaden substrates and microbes (including fungi/viruses/biofilms), test multi-stress durability with realistic soils, deepen characterization and ROS mechanisms, quantify release and life-cycle risks, and run pilot-scale trials with defined QC gates. Although cotton was the primary substrate, our protocol is readily extendable to PET, PET/cotton blends, and PP nonwovens; generating full comparative datasets will clarify substrate-dependent adhesion and wash durability. Broadening the microbial panel (*e.g*., MRSA, Candida) and incorporating antiviral assays (ISO 18184) will better align the platform with medical-textile applications. Mechanistic insight can be deepened *via* operando spectroscopy under UV-A and longitudinal tracking of wash-induced micro-morphology, linking interfacial chemistry to retained photocatalytic performance.

## CONCLUSION

Within the scope of this feasibility study, reclaimed TiO_2_ spray-coating waste—after purification and reconstitution—enabled a roll-to-roll antimicrobial finish on cotton that delivered ≥99.8% bacterial reduction against *E. coli* and *S. aureus* per ISO 20743:2013 and retained high efficacy after 40 laundering cycles. SEM/EDS confirmed the continued presence of TiO_2_ on fiber surfaces, consistent with the observed durability. These findings indicate that waste-derived TiO_2_ can provide practical, scalable antibacterial functionality while potentially reducing reliance on virgin TiO_2_ and diverting industrial waste. The interpretations are limited to the tested fabric, organisms, and laundering protocol; further work should expand the pathogen panel (including fungi/viruses), evaluate fabric performance attributes (hand feel, breathability), and quantify environmental and cost benefits *via* material-balance and life-cycle analyses, alongside direct comparison to virgin TiO_2_ controls. We demonstrate the feasibility of reusing TiO_2_ spray-bath waste to finish antimicrobial textiles *via* roll-to-roll processing while achieving standardized antimicrobial efficacy and wash durability. Surface morphology by SEM confirms uniform coverage consistent with the observed activity, supporting process compatibility without reliance on fresh nanomaterials. This closed-loop approach reduces material demand and effluent, aligning with sustainable manufacturing. Limitations include the absence of batch-resolved phase/chemical-state analytics (XRD/XPS) and broader, real-world durability and release assessments; these will be addressed in future work. Overall, the results indicate a practical pathway to valorize TiO_2_ waste streams into effective antimicrobial finishes at a manufacturing-relevant scale.

## CURRENT & FUTURE DEVELOPMENTS

Looking ahead, broader adoption of this sustainable coating technology could support the textile industry’s transition toward more environmentally responsible production methods. Future research should explore optimizing coating formulations to enhance efficacy under various lighting conditions, integrating the process with AI-enabled quality control for better manufacturing precision, and evaluating performance against a wider spectrum of pathogens. Such developments would further strengthen the role of TiO_2_-based functional textiles in public health protection and sustainable manufacturing.

## AUTHORS’ CONTRIBUTIONS

The author confirms sole responsibility for the following: study conception and design, data collection, analysis and interpretation of results, and manuscript preparation.

## Figures and Tables

**Fig. (1a):**
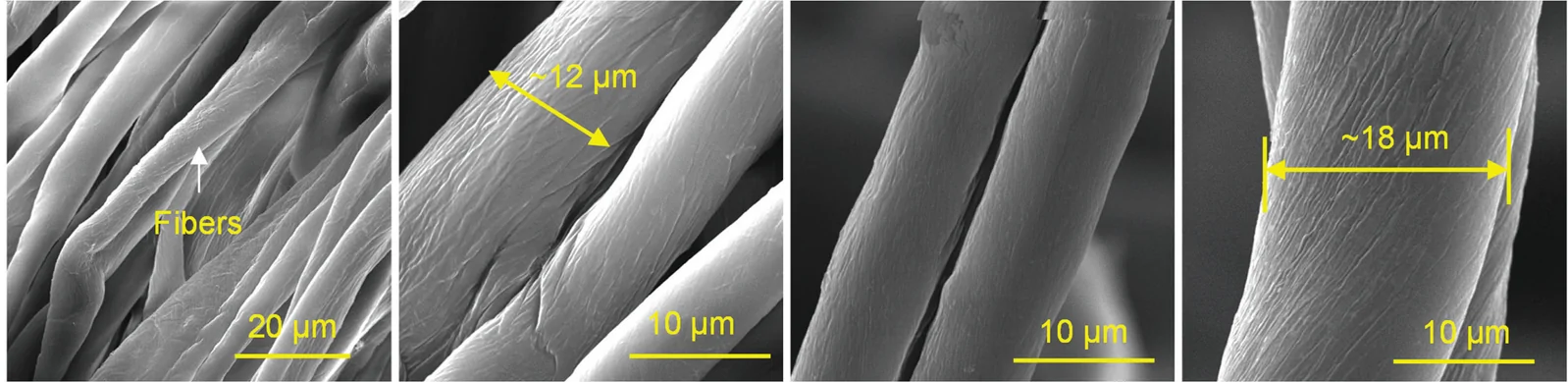
SEM images of uncoated cotton fabrics (fiber dia. < 20 µm). Untreated cotton fabrics (different magnifications; x2000 & x5000.

**Fig. (1b):**
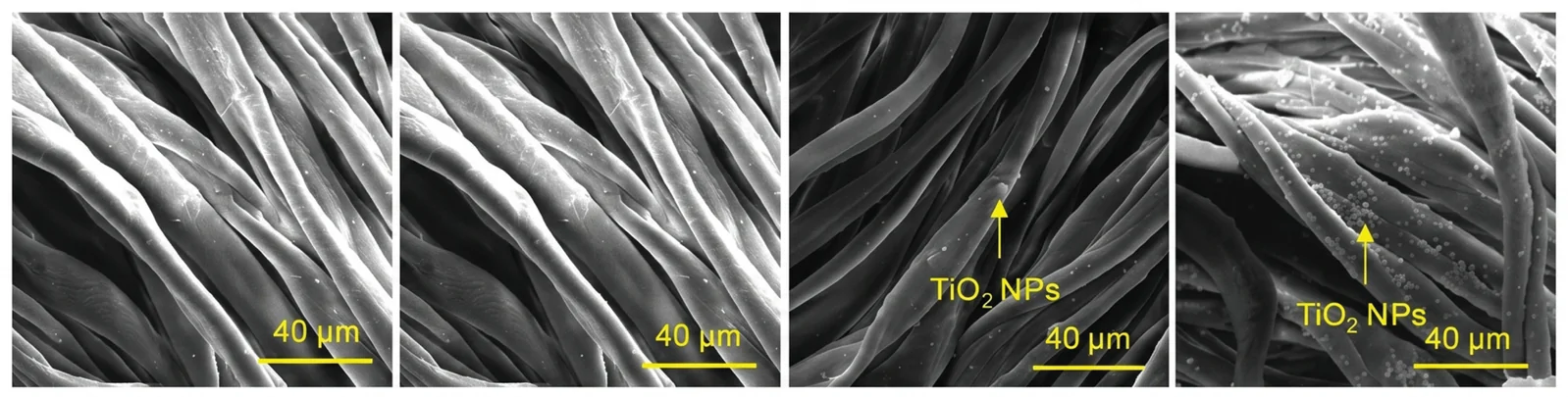
SEM images of coated antimicrobial cotton fabrics (fiber dia. < 20 µm). Treated with a solution of reused waste of TiO_2_ NPs (Magnification: x1000).

**Fig. (4):**
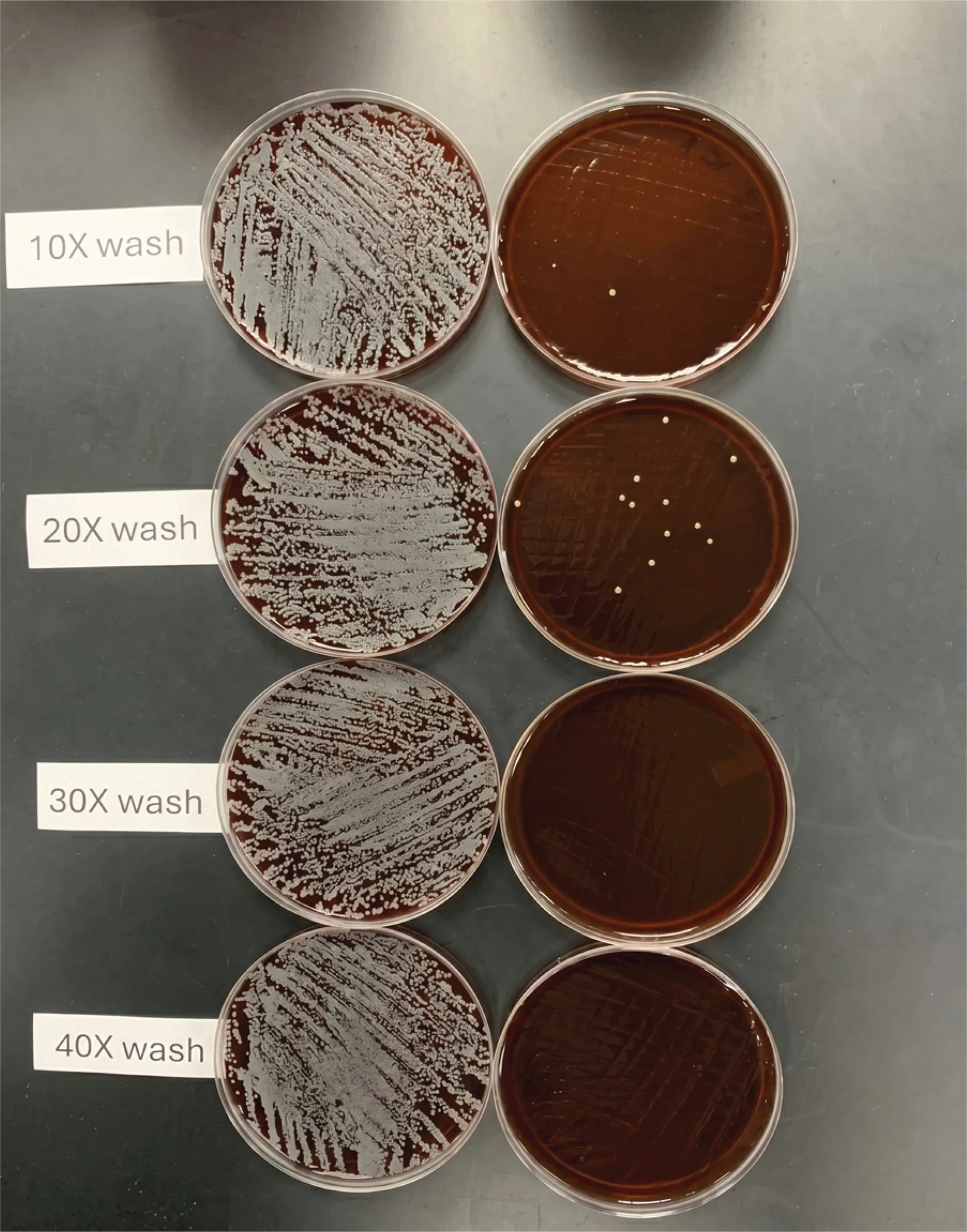
Control *versus* treated washed fabrics exposed to *S. aureus (S.aureus ATCC 29213)* “24-h incubation”. Antibacterial property validated through the outcome of the culture test.

**Fig. (5):**
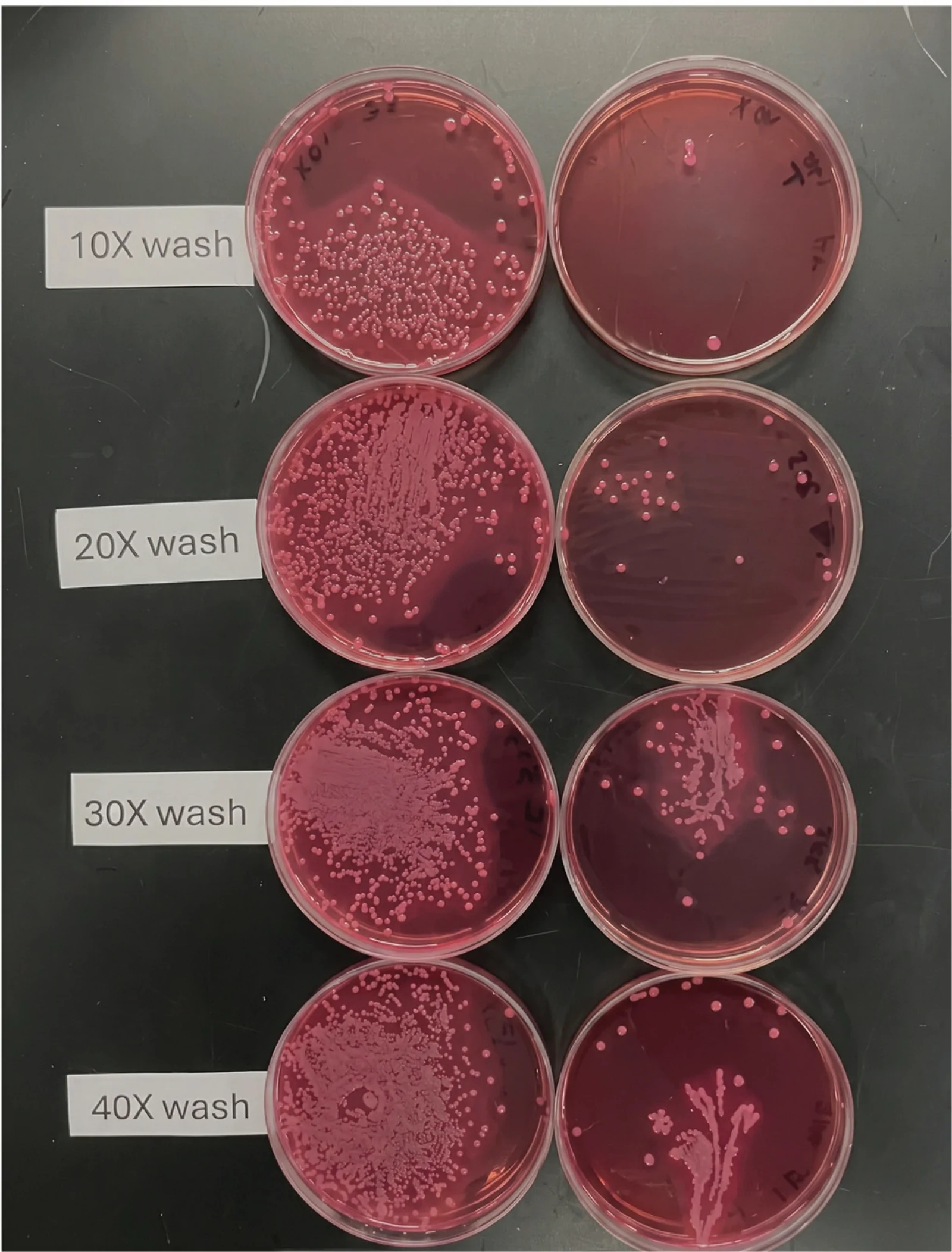
Control *versus* treated washed fabrics exposed to *E. Coli (E.Coli ATCC 25218*) “24-h incubation”. Antibacterial property validated through the outcome of the culture test.

**Table 1:** Antibacterial efficiency of TiO_2_-coated fabrics.

**Bacteria**	**Reduction (%)**
*E. coli*	>99.8
*S. aureus*	>99.8

**Table 2:** Antibacterial efficiency after washing cycles.

**Washing Cycles**	***E. coli* Reduction (%)**	***S. aureus* Reduction (%)**
0	99.99	99.99
10	99.76	99.87
20	99.26	99.30
30	98.73	98.73
40	98.52	98.52

## Data Availability

The datasets generated and/or analyzed during this study are not publicly available but can be requested from the corresponding author.

## LIST OF ABBREVIATIONS

EDS/EDX: Energy Dispersive X-ray Spectroscopy
NPs: Nanoparticles
R2R: Roll-to-roll
ROS: Reactive oxygen species
SEM: Scanning Electron Microscopy
